# Overexpression or absence of calretinin in mouse primary mesothelial cells inversely affects proliferation and cell migration

**DOI:** 10.1186/s12931-015-0311-6

**Published:** 2015-12-22

**Authors:** Walter Blum, László Pecze, Emanuela Felley-Bosco, Beat Schwaller

**Affiliations:** Unit of Anatomy, Department of Medicine, University of Fribourg, Route Albert-Gockel 1, CH-1700 Fribourg, Switzerland; Laboratory of Molecular Oncology, University Hospital Zürich, Labor 40E, Sternwartstrasse 14, 8091 Zurich, Switzerland

**Keywords:** Calretinin, Primary mesothelial cells, Mesothelium

## Abstract

**Background:**

The Ca^2+^-binding protein calretinin is currently used as a positive marker for identifying epithelioid malignant mesothelioma (MM) and reactive mesothelium, but calretinin’s likely role in mesotheliomagenesis remains unclear. Calretinin protects immortalized mesothelial cells in vitro from asbestos-induced cytotoxicity and thus might be implicated in mesothelioma formation. To further investigate calretinin’s putative role in the early steps of MM generation, primary mesothelial cells from calretinin knockout (CR−/−) and wildtype (WT) mice were compared.

**Methods:**

Primary mouse mesothelial cells from WT and CR−/− mice were investigated with respect to morphology, marker proteins, proliferation, cell cycle parameters and mobility in vitro. Overexpression of calretinin or a nuclear-targeted variant was achieved by a lentiviral expression system.

**Results:**

CR−/− mice have a normal mesothelium and no striking morphological abnormalities compared to WT animals were noted. Primary mouse mesothelial cells from both genotypes show a typical “cobblestone-like” morphology and express mesothelial markers including mesothelin. In cells from CR−/− mice in vitro, we observed more giant cells and a significantly decreased proliferation rate. Up-regulation of calretinin in mesothelial cells of both genotypes increases the proliferation rate and induces a cobblestone-like epithelial morphology. The length of the S/G_2_/M phase is unchanged, however the G_1_ phase is clearly prolonged in CR−/− cells. They are also much slower to close a scratch in a confluent cell layer (2D-wound assay). In addition to a change in cell morphology, an increase in proliferation and mobility is observed, if calretinin overexpression is targeted to the nucleus. Thus, both calretinin and nuclear-targeted calretinin increase mesothelial cell proliferation and consequently, speed up the scratch-closure time. The increased rate of scratch closure in WT cells is the result of two processes: an increased proliferation rate and augmented cell mobility of the border cells migrating towards the empty space.

**Conclusions:**

We hypothesize that the differences in proliferation and mobility between WT and CR−/− mesothelial cells are the likely result from differences in their developmental trajectories. The mechanistic understanding of the function of calretinin and its putative implication in signaling pathways in normal mesothelial cells may help understanding its role during the processes that lead to mesothelioma formation and could possibly open new avenues for mesothelioma therapy, either by directly targeting calretinin expression or indirectly by targeting calretinin-mediated downstream signaling.

## Background

Mesothelial cells cover the serous cavities (pleura, pericardium and peritoneum) and surfaces of internal organs. The mesothelial cell monolayer forming the boundary of the tunica serosa to the e.g. intrapleural space is characterized by flattened cells with “cobblestone-like” polygonal morphology. This cell layer has mainly a protective function and serves to lubricating the space between the parietal and visceral serosa, thus reducing mechanical friction. Additionally, the mesothelial cell layer is involved in many other processes including transporting fluid and cells across the serosal cavities, tissue repair and inflammation, coagulation and fibrinolysis and even tumor cell adhesion [1]. Only about 0.15–0.5 % of mesothelial cells undergo mitosis at a given moment in situ; when stimulated e.g. by injury of the serosa, when loosing contact inhibition or through mediators from inflammatory and injured cells, 30–80 % of the mesothelial cells located around the injured site re-enter the cell cycle, i.e. they start synthesizing DNA and proliferate [1, 2].

Calretinin (CR; human gene symbol: *CALB2*) is a Ca^2+^-binding protein of the EF-hand family reported to be absent in normal mesothelial cells in situ; however in reactive mesothelial cells [3] and in malignant mesothelioma (MM), often resulting from asbestos exposure, CR expression is upregulated [3]. Consequently the presence of CR, besides the later discovered mesothelin [1, 4] is viewed as one of the few positive markers for MM, mostly of the epithelioid and mixed type [2, 3, 5]. CR is essential for human MM-derived cell lines in vitro [6], since its down-regulation by shRNA directed against *CALB2* mRNA results in decreased proliferation and significantly reduced viability, the latter mostly caused by induction of apoptosis via activation of the intrinsic caspase 9-dependent pathway. Down-regulation of CR in immortalized (non-transformed) human mesothelial cells (e.g. LP-9/TERT1) results in a G_1_ growth arrest without leading to apoptosis or necrosis [6]. Impairment of Ca^2+^ handling in MM cells reduces uptake of Ca^2+^ into mitochondria and this reduces apoptosis in these cells [7]. In line with this study, overexpression of CR reduces the mitochondrial Ca^2+^ uptake in primary mesothelial cells [8].

In order to further investigate the role of CR in cells of mesothelial origin, we made use of mouse-derived primary mesothelial cells from wild-type (WT) mice and from CR-deficient (CR−/−) animals. We observed that CR−/− cells displayed reduced cell proliferation and decreased mesothelial cell layer regeneration (scratch assay in vitro), while CR overexpression increased cell proliferation and mobility in both genotypes.

## Methods

### Isolation of mesothelial cells

Mesothelial cells were isolated from 4–6 months old C57Bl/6 J mice (WT) and from CR−/− mice also on a C57Bl/6 J background; the detailed cell isolation procedure is described elsewhere [9, 10]. All experiments were performed with permission of the local animal care committee (Canton of Fribourg, Switzerland) and according to the present Swiss law and the European Communities Council Directive of 24 November 1986 (86/609/EEC). Briefly, mice were sacrificed and the peritoneal cavities were exposed by incision. The peritoneal cavities were washed by injection of approximately 50 ml of PBS (Sigma, St. Louis) via a 25G x 5/8” needle (BD microlance 3, Becton Dickinson AG, Allschwil, Switzerland) using a peristaltic pump and a second needle to allow exit of the PBS solution. Perfusion was maintained until the exiting PBS solution was clear, i.e. devoid of mobile and poorly attached cells. Residual PBS was aspired with a syringe and the peritoneal cavity was filled with 5 ml of 0.25 % Trypsin/EDTA solution (Gibco, Switzerland). The body temperature of mouse corpses was maintained at around 37 °C for 5 minutes via an infrared heat lamp. The suspension containing the detached cells was collected with a syringe, cells were centrifuged for 10 min at 300 x g. Cells mostly comprising primary mesothelial cells were grown in modified Connell’s Medium composed of: DMEM/F12 + GlutaMax (Gibco), 15 % FCS, 0.4 μg/ml hydrocortisone, 10 ng/ml epidermal growth factor, 1 % ITS (insulin, transferrin, selenium), 1 mM sodium pyruvate, 0.1 mM beta-mercaptoethanol, 1 % non-essential amino acids, 1 % Penicillin-Streptomycin and 2 % Mycokill (PAA, Brunschwig, Switzerland) [11]. All animals were genotyped by PCR using the forward primer CR-IT1 (5’ common primer) 5’-GCTGGCTGAGTACTCCAAGGGTACACATT-3’ and the reverse primer 5’-GTTCTCTAGCTCTTTACCTTCAATGTACCCCA-3’ for the WT *Calb2* allele (fragment size of 243 bp) and the reverse primer 5’-GTCTCCGTGGAGGTGGTGACTTCCTAGTC-3’ for the mutated *Calb2* allele (fragment size of 150 bp).

### Hematoxylin and eosin staining

WT and CR−/− mice were killed by CO_2_ inhalation followed by intracardial perfusion with PBS. The tissue was fixed by perfusion with 4 % paraformaldehyde (PFA) for 10 min and post-fixation by immersion in the same solution. Small pieces of various tissues including lung, large and small intestine were dissected, embedded in paraffin and semi-thin sections (10 μm) were prepared and stained with hematoxylin and eosin solution. Primary mesothelial cells were grown on glass coverslips in 6-well plates until confluence was reached and fixed with 4 % PFA and stained with hematoxylin and eosin.

### Transmission electron microscopy

Fifty thousand primary mesothelial cells were seeded on PET track-etched membranes with 3-μm pores (Becton Dickinson AG, Allschwil, Switzerland) and grown for 96 h in Connell’s Medium. Then, cells were fixed with 2 % PFA/ 2.5 % glutaraldehyde solution for 90 min and processed as described before [12, 13]. Images were taken with a Philips CM100 Biotwin transmission electron microscope and acquired with the iTEM software (Olympus Soft Imaging Solutions GmbH, Germany).

### Plasmids, transfections and lentivirus production

CR was overexpressed by using the lentiviral system pLVTHM (Addgene plasmid #12247). Briefly the GFP cassette in pLVTHM was replaced with human *CALB2* cDNA coding for full-length CR using the expression plasmid CMV-CALB2-neo as template [14]. The required cDNA fragment coding for full-length CR was synthesized by PCR using the primers PmeI-CALB2 (5’-AGT CGT TTA AAC ATG GCT GGC CCG CAG CAG CAG-3’) and SpeI-CALB2 (AGT CAC TAG TTT ACA TGG GGG GCT CGC TGC A-3’). The amplicon was digested with PmeI and SpeI and inserted into the unique sites of the pLVTHM vector to produce the final pLV-CALB2 plasmid. The pCMV/TO SV40 large + small T Antigen plasmid was obtained from Addgene (#22298). pLKO.1-shCALB2 #7 (shRNA *Calb2* sequence: 5’CCG GGA AGG AGT TCA TGC AGA AGT ACT CGA GTA CTT CTG CAT GAA CTC CTT CTT TTT G-3’) and pLKO.1 H2B-GFP (Addgene # 25999) and lentivirus production was carried out as described before [6]. The pLV-NLS-CR plasmid was created using the following plasmids as template: CMV-CR-neo for CR and pLV-EBFP2-nuc (Addgene #36085) encoding a nuclear localization signal (NLS). The required cDNA fragment coding for CR was synthesized by PCR using the following primers 5’-GAG ACT CGA GTA GCT GGC CCG CAG CAG C-3’ and 5’-GAG ATC TAG ATT ACA TGG GGG GCT CGC TGC A-3’ and integrated into the pGEM-T Easy vector (Promega, MA, USA) using the TA ligation method. The PCR product for NLS was amplified with the following primer pairs: 5’-GAG ACC ATG GGT TTA AAC ATG GCT GAT CCA AAA AAG AAG AGA AAG-3’ and 5’-GAG ACT CGA GAT CTA GAT CCG GTG GAT CC-3’. The amplicon containing the NLS sequence was digested with AatII and NcoI and inserted into the appropriate site of pGEM-T Easy-CALB2 to generate pGEM-t-Easy-NLS-CR plasmid. Then, the NLS-CALB2 sequence was excised with PmeI and SpeI and integrated into the pLVTHM vector to produce the final pLV-NLS-CR plasmid.

### Cell growth and viability assays

250**–**1000 cells/well were seeded in 96-well plates in Connell’s medium and grown for 7 days. Cell confluence was measured using the Live Cell Imaging System (Incucyte, EssenBioScience, Michigan, USA) by acquiring images every 3 h. As a complementary method, cell number/viability was assessed at selected time points by performing an MTT assay as described before [6]. Cells were seeded in 24-well plates at a confluence of about 50 % and transfected 24 h later with 1 ml of non-concentrated lentivirus suspension containing Polybrene (8 μg/mL; Sigma, Buchs, Switzerland). Cells were grown for 3 days, passaged at a dilution of 1:8 and cell proliferation was monitored with the Incucyte system.

### Cytotoxicity assay

Cells were plated at a density of approximately 10 % and the presence of dying cells was monitored in the Incucyte system using the CellToxGreen dye (Promega, San Luis Obispo, CA, USA) following the manufacturer’s instruction for real-time cytotoxicity analysis. The image series were evaluated and the dying cells were determined with the particle analysis function of ImageJ software (NIH, USA).

### Western blot analysis

Protein samples were isolated from cultured mouse mesothelial cells and from mouse tissue. Cells were grown in 25 cm^2^ flasks and harvested at confluence. Cytosolic protein fractions were collected as described before [15]. Freshly excised mouse cerebellum was frozen in liquid nitrogen and homogenized in extraction buffer (10 mM Tris, 2 mM EDTA, 1 mM β-mercaptoethanol; pH 7.4) containing a cocktail of different protease inhibitors (Roche, Mannheim, Germany). Proteins (100 μg) from each cell culture sample, 1 μg protein from cerebellum, as well as 40 ng of purified human recombinant CR were loaded onto SDS-polyacrylamide gels (10 %). After separation, proteins were blotted onto a nitrocellulose membrane (Bio-Rad Laboratories, Hercules, CA, USA) and incubated for two days at 4 °C with the CR-specific antibody CR7699/4 (Swant*,* Marly*,* Switzerland) at a dilution of 1:10,000. Rabbit secondary antibody linked to horseradish peroxidase (Sigma-Aldrich) was diluted at 1:10,000 and incubated for 2 days; prolonged incubation was shown to enhance the sensitivity in Western blotting [16]. For the detection, the chemiluminescent reagent Luminata Classico Forte (EMD Millipore Corporation, Billerica, MA, USA) was used. Chemiluminescent and normal illumination digital images were recorded on a system from Cell Biosciences (Santa Clara, CA, USA).

### Immunohistochemistry (IHC)

Cells were seeded on glass coverslips and incubated with the appropriate medium until a confluence of 70–90 % was reached. Then, the cells were fixed with 4 % PFA, permeabilized with 1 % Triton X-100, and blocked with TBS-containing donkey serum (10 %). Cells were incubated overnight at 4 °C with the primary antibodies at the indicated dilutions: rabbit polyclonal anti-CR (Swant, 7699/4; 1:500), mouse monoclonal anti-mesothelin (Santa Cruz sc-166203; 1:500), mouse monoclonal anti-pan-cytokeratin, clone Lu-5 (BMA Biomedicals, T*-*1302; 1:500), mouse monoclonal anti-vimentin (Sigma V6630; 1:500) or rabbit polyclonal anti-desmin (Sigma D8281; 1:500). After washing, the cell-containing coverslips were incubated with secondary antibodies for 1 h with either DyLight488-conjugated donkey anti-mouse IgG (Jackson Immunoresearch Laboratories; 1:1000) or Cy3-conjugated donkey anti-rabbit IgG (Jackson Immunoresearch Laboratories; 1:1000). The cells were counterstained with DAPI (Molecular Probes; 5 μg/ml) and mounted with Hydromount solution (National Diagnostics, Atlanta, GA). Images were acquired with a LEICA fluorescent microscope DM6000B (Wetzlar, Germany*)* integrated to a Hamamatsu camera C4742-95 (Bridgewater, New Jersey, USA).

### Scratch assay

Mesothelial cells (10,000–20,000) were plated in a 96-well ImageLock plates (Essen Bioscience) and 24 h after plating, a scratch of about 1 mm was created using the Wound Maker tool (Essen Bioscience) and the cell culture medium was replaced. The plate was scanned at a 2 h-frequency and data was evaluated using the Incucyte Software. Primary mesothelial cells were transduced with lentivirus expressing pLKO.1 H2B-GFP coding for a fusion protein consisting of histone 2B fused to green fluorescent protein and mixed at a ratio of 1:1; the scratch area was monitored every 15 min using the live cell imaging system (Incucyte FLR 10x, Essen Bioscience). To assess the cell mobility, the movements of the cells migrating primarily towards the center of the scratch were recorded. For the image analysis a Java ImageJ plugin (CGE) was used (http://bigwww.epfl.ch/sage/soft/circadian/) that had been developed for tracking cells in the context of circadian studies [17].

### Bioluminescence time-lapse microscopy and data analysis

To monitor the cell-cycle, cell division and cell movement, GFP-labeled primary mesothelial cells from CR−/− and WT mice were infected with a lentivirus coding for mCherry-hCdt1 (30/120) [18, 19]. The Fucci (Fluorescent, ubiquitination-based cell cycle indicator) mCherry-hCdt1 was a kind gift of Prof. H. Miyoshi (Riken, Japan). mCherry-hCdt1 was synthetized by PCR with the forward primer (FW-PmeI-mCherry 5’-AGT CGT TTA AAC ATG GTG AGC AAG GGC GAG GAG-3’) and reverse primer (RV-SpeI-mCherry 5’-AGT CAC TAG TTT AGA TGG TGT CCT GGT CCT G-3’) and cloned into pLVTHM (Addgene plasmid #12247) (SpeI and PmeI sites) substituting eGFP. pLV-mCherry-hCdt1 was used to produce lentivirus. The expression level of mCherry-hCdt1 is cell cycle-dependent showing an accumulation during the G_0_/G_1_ phase, followed by an ubiquitination*–*based protein degradation during S/G_2_/M phases. Fluorescence and time*-*lapse microscopy was performed with an inverted fluorescence microscope DMI 6000B (Leica Microsystems) equipped with an incubation chamber. A digital camera (Leica), a 10× objective, GFP and TXR filter sets and the LAS-AF imaging software (Leica) were used to acquire the images. Images were taken every hour using the same settings including exposure times, gains and positions. For the image analysis a Java ImageJ plugin: CGE [17] was used.

### Statistical analysis

Two-tailed t-tests were implemented in Excel 2010 (Microsoft). Linear regressions were fitted to the experimental data from scratch assays using the GraphPad Prism (GraphPad Software, San Diego, California, United States) analysis package. MTT results were averaged (n ≥ 3 independent experiments, each sample (experimental condition) at least measured in triplicates). The statistical significance was calculated using a one-way ANOVA with StatPlus (AnalystSoft).

### Immunohistochemistry on sections of mouse embryos from E14.5 and E16.5

Embryos from C57Bl/6J mice were collected at embryonic day 14.5 (E14.5) and 16.5 (E16.5). Embryos were fixed by immersion in 4 % PFA for 72 h and embedded in paraffin after dehydration. De-paraffinized sections (3 μm) were subjected to Tris/EDTA (1 mM/0.1 mM, pH 9) antigen retrieval by heating the sections in a boiling water bath for 20 min. Endogenous peroxidases were quenched in 0.3 % H_2_O_2_ for 20 min followed by tissue permeabilization in 0.1 % PBS Tween 20 for 5 min and blocked at room temperature for 20 min in PBS containing 2 % BSA and 1 % horse serum. Sections were incubated with primary antibodies (anti-CR 7699/4, Swant, Switzerland) 1:200 overnight at 4 °C. Sections were washed and incubated with secondary antibodies (1:200) at room temperature for 2 h. DAB (3,3’-diaminobenzidine tetrahydrochloride (Sigma-Aldrich) staining was performed followed by counterstaining of sections with hematoxylin. Slides were scanned and analyzed using a whole slide imaging system from Hamamatsu (Nanozoomer, 2.0-HT).

## Results

### Growth characteristics of mouse primary mesothelial cells in vitro

Primary mesothelial cells (prMC) isolated from the mouse peritoneum showed the typical growth characteristics as most primary cells. Directly after isolation, prMC cells grew rather slowly in vitro, the likely result of the adaption phase to the novel situation, e.g. with respect to growth medium, cell attachment surface, etc. After few passages, cell proliferation increased evidenced by the shortening of the time required to reach confluence and steady-state rate of growth (maximum proliferation rate) was generally observed between passages 2–6. At later passages (n > 10), prMC started to show signs of senescence evidenced by the decrease in proliferation rates. For the reason of variable prMC proliferation characteristics in vitro, in all experiments parallel cultures (isolated from age-matched WT and CR−/− mice at the same day) having an identical number of passages at the start of the experiment were used. Also in overexpressing experiments, where CR or the nuclear-targeted NLS-CR was expressed in prMC, the appropriate control cells were treated at the same time point by exactly the same procedure. Thus, growth curves presented in this study are variable to some extent, e.g. with respect to the absolute time required to reach confluence. For these reasons, growth curves were evaluated in relative (side-by-side comparison), not in absolute terms.

### The tunica serosa including the mesothelial cells from CR-deficient (CR−/−) mice is indistinguishable from the one of WT mice

**Fig. 1:**
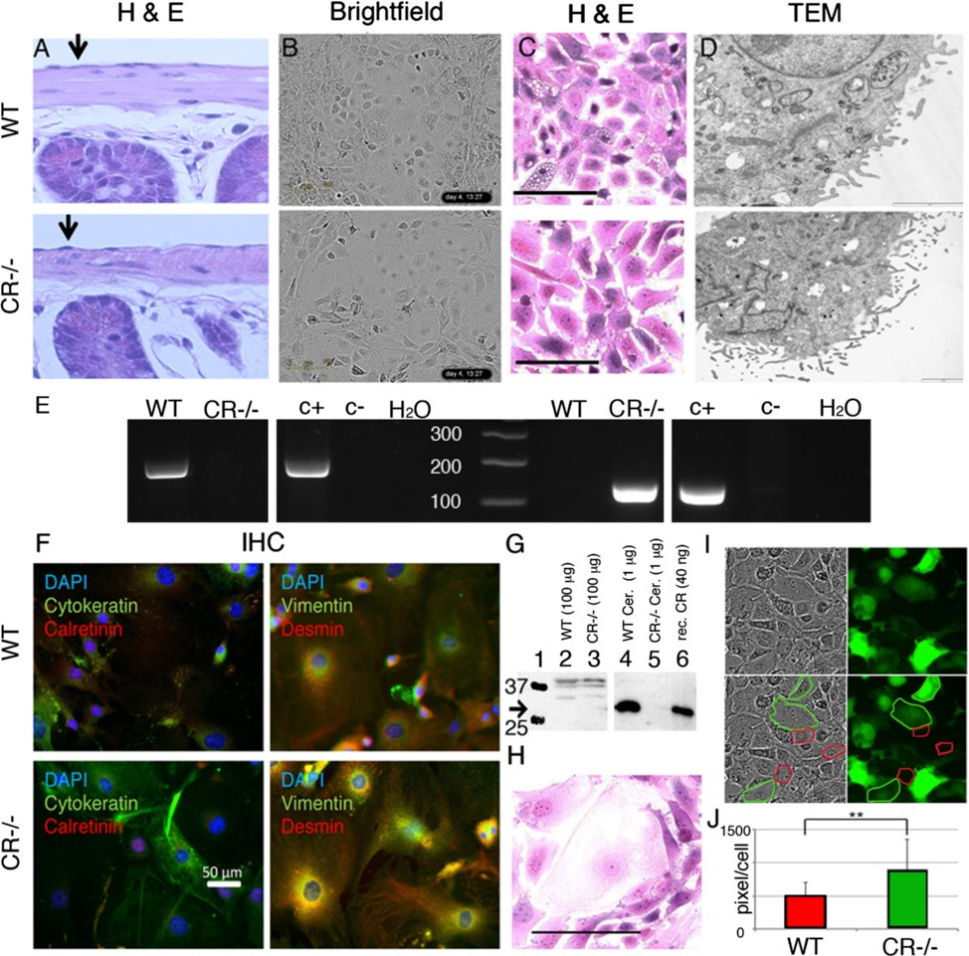
**a** Hematoxylin and Eosin (HE) staining of a longitudinal section of small intestine from a WT and CR−/− mouse showing a normal mesothelium. Primary mesothelial cells of both genotypes grown in vitro maintain their typical ‘cobblestone-like’ morphology, when grown to confluence as evidenced on the brightfield images (**b**). **c** HE staining of primary mesothelial cells grown in vitro revealed that CR−/− cells tended to be more flattened (larger diameter) and more “giant” cells (largest diameter > 100 μm) were observed, a typical example is shown in (**h**) Scale bar = 100 μm. The TEM pictures (**d**) demonstrate the presence of microvilli typical for mesothelial cells; no apparent differences with respect to microvilli number, size or length were noticed. **e** Genotyping of WT and CR−/− mice with primers recognizing the *Calb2* WT allele (*left panel*) and the mutated allele (*right panel*). For each PCR analysis, a positive control (c+) and a negative control (c-) from genomic DNA of previously identified WT and CR−/− mice was amplified, as well as a PCR reaction without template DNA (H_2_0 control). **f** Immunofluorescence images of primary mesothelial cells in vitro stained for cytokeratin and calretinin (*left*) and for vimentin and desmin (right), derived from WT (*upper panel*) and CR−/− (*lower panel*) mice. Nuclei are stained with DAPI (*blue*). Note the presence of all three intermediate-filament markers and the absence of a specific CR signal in cells from both genotypes. **g** Western blot analysis of CR in protein extracts from cerebellum (lanes 4 & 5) and prMC grown in vitro (lanes 2 & 3) from WT (2 & 4) and CR−/− (3 & 5) mice. Molecular weight markers (25 and 37 kDa) are shown in lane 1, purified recombinant CR (control) is shown in lane 6. Upper bands (>40 kDa) in lane 2 and 3 are non-specific bands not corresponding to the size of CR. **i** Mixed population of WT and CR−/− EGFP-labeled mesothelial cells: note the increase in cell surface area covered by a single CR−/− mesothelial cell (green boundaries) compared to WT cells (red boundaries). **j** Quantification of the average surface area covered by a WT or a CR−/− prMC is shown (*n* = 25 cells for CR−/− and *n* = 24 cells for WT, ** *p* < 0.01). For the analysis giant cells were omitted

The tunica serosa facing the peritoneal cavity and covering the small intestine from WT and CR−/− mice was compared (Fig. 1a). In both cases a single layer of flat mesothelial cells characterized by a disk-shaped nucleus formed the barrier towards the peritoneum. Neither signs for a change in mesothelial cell morphology (e.g. cuboidal cells), thickening of the tunica serosa nor other abnormalities were detected in CR−/− mice. Also in the pleural mesothelium, there were no indications of an altered mesothelial cell layer (data not shown). Isolated primary mesothelial cells were incubated in Connell’s medium and let grow for approximately 1 week until confluence was reached. Starting at a confluence of about 10 %, primary mesothelial cells completely covered the surface of the plates after a growth period of 2–4 days in Connell’s medium. At confluence, mesothelial cells from either genotype showed the typical cobblestone-like morphology, demonstrated contact inhibition and stopped growing. Brightfield images of mesothelial cells are shown in Fig. 1b. When starting with an identical initial cell density (500 cells per 96-well), CR−/− cells tended to grow slower than the corresponding WT cells. This was also evident when the surface covered by an individual mesothelial cell was compared (Fig. 1b, c, i). At confluence, the number of mesothelial cells per surface was smaller also evidenced by a more pronounced cell flattening in the CR−/− samples hinting towards a reduced rate of cell proliferation. We observed a higher prevalence of giant cells (Fig. 1h) in cultures derived from CR−/− mice.

The identity of the cells grown in vitro as mesothelial cells was additionally revealed by TEM images. We found the typical hallmark for mesothelial cells, i.e. rather short microvilli of variable sizes that are not present in lymphocytes or fibroblasts (Fig. 1d), which might have been co-isolated with the mesothelial cells. We found no qualitative evidence for significant changes in the size distribution and amount of microvilli between CR−/− and WT cells. Furthermore, mesothelial cells were identified by their expression of specific intermediate filaments; WT and CR−/− cells expressed both epithelial (e) and mesenchymal (m) intermediate filaments including cytokeratin (e), vimentin (m) and desmin (m) (Fig. 1f) as described before [20]. Genotyping of mice used for primary mesothelial cell isolation was done by PCR using allele-specific primers (Fig. 1e). Finally, we investigated the presence of CR protein expression in primary mouse mesothelial cells. Western blot analysis control experiments with proteins isolated from WT and CR−/− cerebellum revealed a strong signal in the WT sample with an identical size as purified recombinant CR, while no signal was seen with cerebellar extracts from CR−/− mice (Fig. 1g). In cellular extracts from primary mesothelial cells, no specific CR signal was detected in cells from both genotypes (Fig. 1g). Moreover, also by immunohistochemistry, no CR-specific signal was detected in WT and CR−/− mesothelial cells (Fig. 1f). This suggested that either CR was absent in WT mesothelial cells or that expression levels were below the detection limit of the Western blot analysis and immunohistochemistry. Although the cobblestone-like morphology of primary mesothelial cells from WT and CR−/− mice was similar, we noticed that CR−/− cells covered a larger surface area (Fig. 1b, c). Thus, CR−/− cells were infected with lentivirus expressing the enhanced green fluorescent protein (eGFP). These cells were then mixed at a 1:1 ratio with WT primary mesothelial cells and grown to confluence (Fig. 1i). From brightfield images and fluorescence images, CR−/− cells (green) and WT cells (non-green) were randomly selected (red and green traces, respectively) and the surface area was determined. The sparse giant cells of both genotypes were excluded from the analyses. The average surface area covered by a typical CR−/− cell was significantly larger (+74.6 %) in comparison to a WT cell (Fig. 1j).

### Mesothelial cells, but not lung fibroblasts from CR−/− mice proliferate significantly slower

**Fig. 2:**
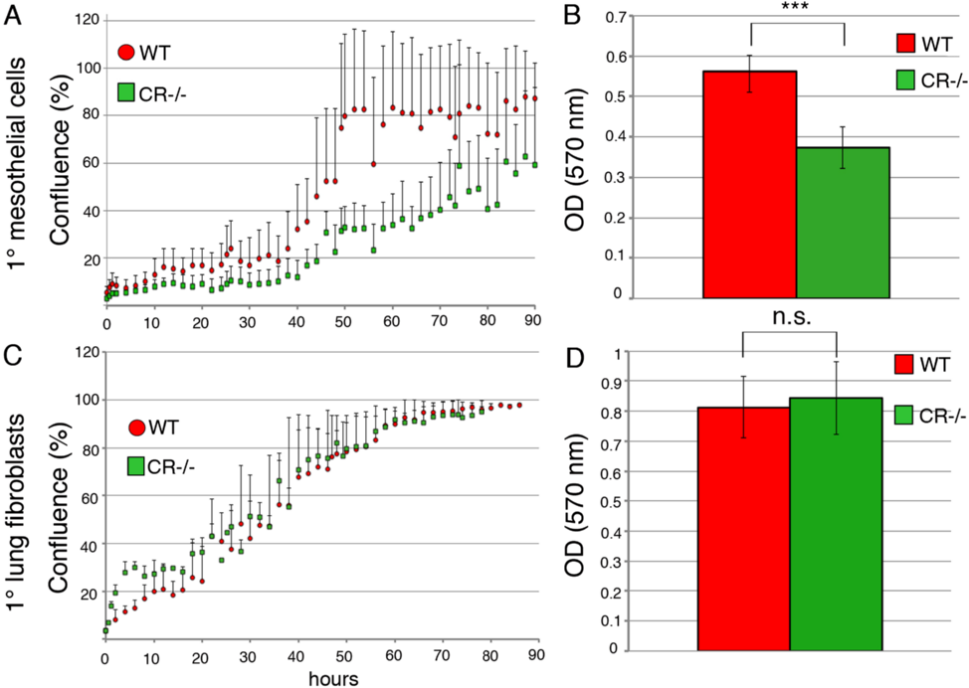
**a** Cell growth analyses (average of five wells, four independent experiments showing similar growth curves) were performed. Primary mesothelial cells show clear differences between WT and CR−/− cells (**a**). A plateau (100 % confluence) is reached at ≈ 50 h in WT cells, while CR−/− cells are not yet confluent (≈60 %) at 90 h. **b** The MTT signal obtained at 96 h post-seeding is significantly smaller in CR−/− samples (*n* = 3 experiments; *p* < 0.005) (**c**) Growth curves and (**d**) MTT signals at 96 h post-seeding from primary lung fibroblasts are not different between genotypes

To further document the differences in primary mesothelial cultures from WT and CR-deficient mice a detailed growth curve analysis was carried out. At a starting confluence of 10 %, 100 % confluence (plateau level) in rapidly proliferating WT cells was reached after approximately 50 h; at the same time point CR−/− cells from parallel cultures showed only approximately 30 % confluence and even after 90 h the plateau was not yet reached (Fig. 2a). Alternatively, we used the MTT assay that reports the combined effects of cell number and metabolic activity [21]. Assuming that under our growth condition, cell metabolism should not be impaired, the MTT readout is expected to be proportional to the cell number. At the selected time point (96 h, endpoint), the MTT signal was significantly smaller for CR−/− than for WT cells supporting the data on reduced CR−/− cell proliferation determined with the live cell imaging system (Fig. 2b). To investigate whether this difference was also observed in another cell type we analyzed primary lung fibroblasts from both genotypes using both assays. Growth curves obtained with the live cell imaging system and MTT values at 96 h post-seeding showed no differences between fibroblasts isolated from WT or CR−/− mice (Fig. 2c, d). Also the overall morphology of fibroblasts from the 2 genotypes was not different (data not shown). This suggests cell type-specific differences in the proliferation of WT and CR−/− cells.

### Upregulation of CR in primary mesothelial cells from WT and CR−/− mice increases proliferation

**Fig. 3:**
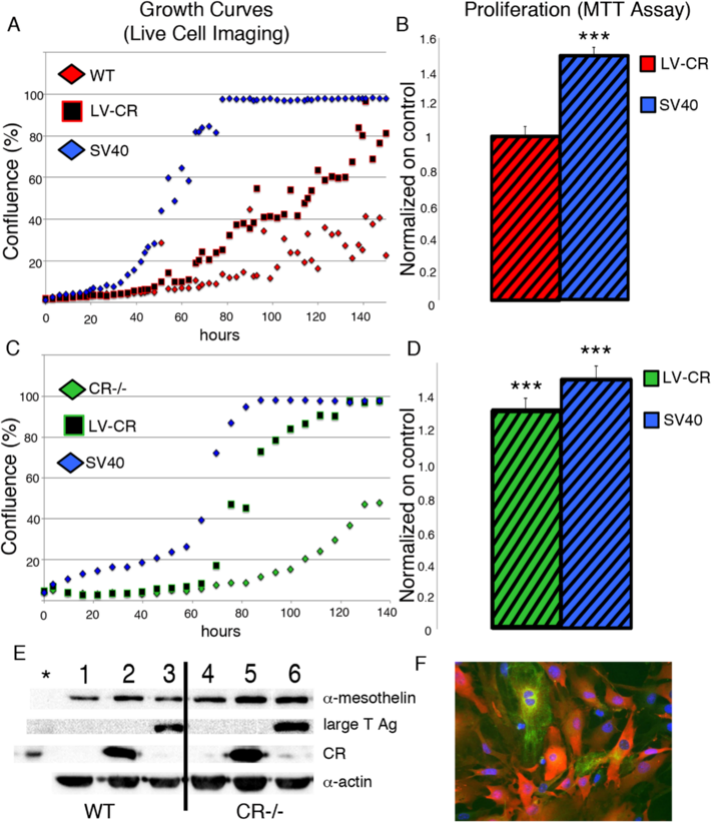
**a** Real-time growth curves of WT prMC (control), cells transfected with a SV40 plasmid (SV40) or a CR expression plasmid (LV-CR). **b** MTT signals at 96 h (normalized to WT cells) are increased in cells expressing SV40 TAg/tag. **c** Growth curves for CR−/− prMC subjected to the same treatment as in (**a**). **d** Normalized MTT signals in CR−/− cells; all experimental details are the same as in (**b**) (*n* = 3, one representative growth curve and MTT experiment is shown). Western Blot analysis in (**e**) show a strong upregulation of CR and SV40 large T Antigen (TAg) in the corresponding cell cultures. α-Mesothelin is used as marker for mesothelial cells and α-actin serves as a loading control. **f** Immunohistochemistry for CR (*red*) in CR-overexpressing WT cells and cytokeratin (*green*), cell nuclei are stained with DAPI (*blue*) (***p < 0.0005)

Primary WT mesothelial cells from later passages (n > 7) tended to grow slower, reaching approximately 40 % confluence at 140 h in culture (Fig. 3a). When these cells were transfected with the CR expression plasmid coding for full-length CR (pLVTHM-CALB2), a strong CR protein signal at the expected size (M_r_: 31 kDa) was observed by Western blot analysis (Fig. 3e) and immunohistochemistry revealed intense and relatively homogenous staining that was strongest in the cytosol (Fig. 3f). No qualitative differences in the staining for the markers cytokeratin, vimentin and desmin were observed in CR-overexpressing mesothelial cells (data not shown). The proliferation rate was clearly increased, i.e. with the same number of initially seeded CR-overexpressing cells, 80 % confluence was reached after 140 h (Fig. 3a). As a positive control, WT cells were transfected with the SV40 plasmid coding for the large T and small t antigen (TAg and tag, respectively); increased TAg and tag expression was previously shown to result in increased proliferation, telomerase activity and favor transformation of mesothelial cells [22, 23]. Under these experimental conditions, TAg/tag-expressing WT mesothelial cells grew even faster, reaching a plateau (100 % confluence) already at approximately 70 h (Fig. 3a). Results of the MTT assays performed at 96 h post-plating showed rather similar results. Compared to WT control cells, overexpression of TAg/tag (SV40) resulted in a clearly higher (+40 %) MTT signal (Fig. 3b). Experiments with CR−/− mesothelial cells resulted in similar results with respect to CR or TAg/tag overexpression. The strongest effect on increasing proliferation was observed after SV40 transfection, but also CR overexpression significantly increased proliferation determined by the 2 assays (live cell-imaging, MTT assay; Fig. 3c, d).

### Decreased proliferation and mobility of CR−/− mesothelial cells leads to a prolongation of the scratch-closure time

**Fig. 4:**
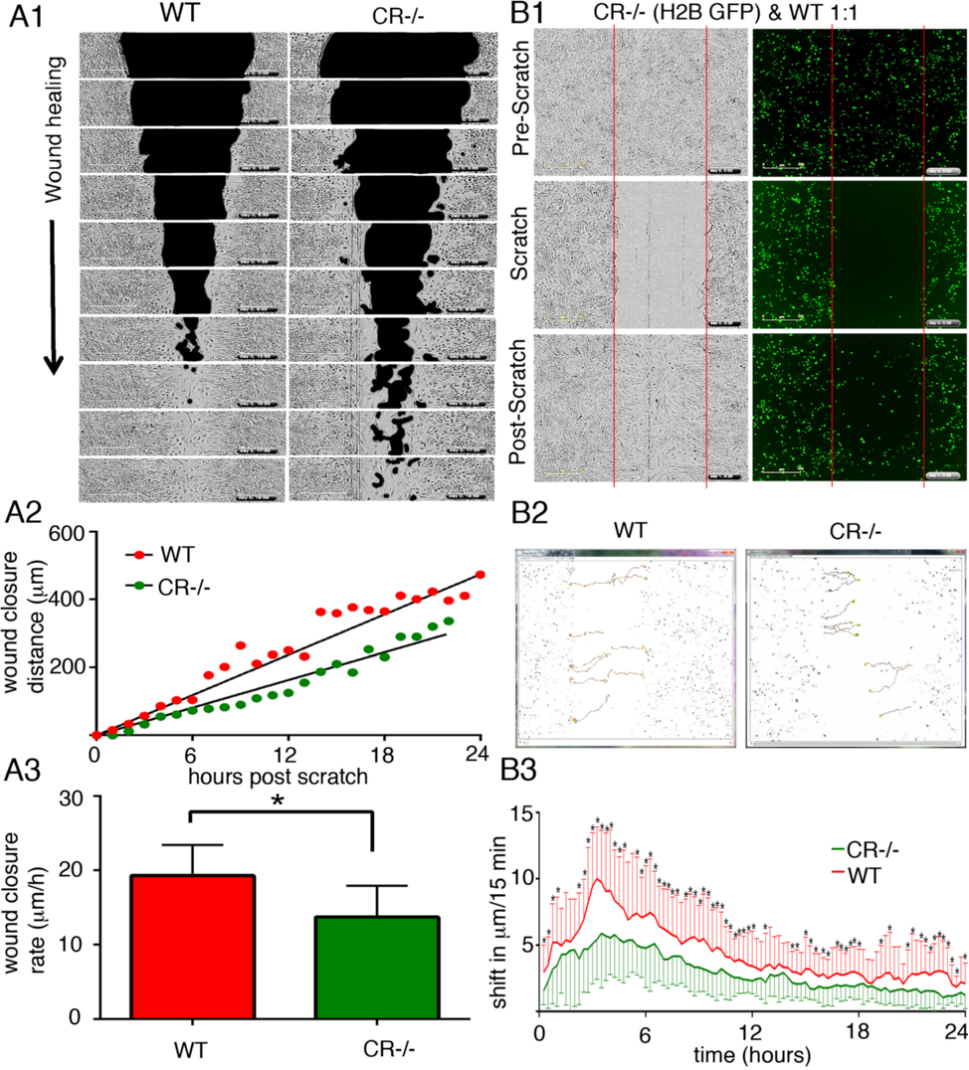
**a1** Time-lapse brightfield images were taken after a scratch (*black area*) was made at t = 0 in a confluent layer (*grey area*) of prMC from WT and CR−/− mice. Images were taken every 2 hours and “wound closure” was measured as the rate at which the scratched area (*black*) was repopulated with mesothelial cells (*grey zone*). **a2** The wound closure distance as a function of time resulted in the rate of cell re-colonization (“wound closure rate”: slope); a representative example for WT and CR−/− prMC is shown. **a3** Quantitative analyses yielded closure rates of 19.3 ± 4.1 μm/h (mean ± S.D.) for WT and 13.7 ± 4.2 μm/h for CR−/− prMC (8–10 independent experiments per genotype; *p < 0.05; t-test) (**b1**) A scratch was made in a confluent layer initially consisting of non-fluorescent (*black*) WT cells and H2B - GFP-labeled CR−/− cells at a ratio of 1:1. The pre-scratch situation is depicted in the upper panel (brightfield image: *left*; GFP fluorescence (*right*). After complete gap closure, the initially void region was mostly filled (invaded) by non-fluorescent WT cells. **b2** Measurement of cell mobility of single mesothelial cells (*n* = 10 cells per genotype) shows significant differences (*p < 0.05 two tailed t-test) in the mobility of WT and CR−/− cells quantified in (**b3**), where the cell movement was measured (in μm) per 15 min time interval

If the mesothelial cell layer is injured in vivo, e.g. by exposure to asbestos fibers, the closing of the injured site is dependent on several factors including the rate of the proliferation of the healthy cells adjacent to the injured site, as well as to cell mobility on the exposed *lamina propria* consisting mostly of extracellular matrix. Such a situation can be mimicked in vitro, at least in part, by the 2D-migration assay (scratch assay). Confluent layers of primary WT and CR−/− mesothelial cells were subjected to a scratch resulting in a gap of approximately 1 mm. The empty space was then filled by a process consisting of proliferation and migration of these newly generated cells. As revealed by time-lapse experiments, closing of the gap occurred clearly faster with WT mesothelial cells than with CR−/− cells (Fig. 4A1). From the scratch-closure distance (proportional to closure area; Fig. 4A2) as a function of time, the slope representing the wound closure rate was calculated. The value was clearly lower in CR−/− cells compared to WT cells (−29 %; Fig. 4A3). H2B (histone 2B)-GFP - labeled CR−/− mesothelial cells were mixed at a ratio of 1:1 with non-fluorescent WT cells and cells were let to grow to confluence (Fig. 4B1). Then, a scratch was made and cells were grown again to 100 % confluence, i.e. until the gap was closed. The gap was colonized to a large extent by non-fluorescent WT mesothelial cells (Fig. 4B1), in line with results shown in Fig. 4A1. Histone-GFP labeled cells were monitored after the scratch to assess cell mobility (Fig. 4B2) and quantified as distance travelled (in μm) per 15 min (Fig. 4B3). CR−/− cells showed a significantly decreased mobility (*p < 0.05).

### Prolongation of the G_1_ phase of CR−/− mesothelial cells is the major cause for the decrease in proliferation and the prolonged gap-closure time in the scratch assay

**Fig. 5:**
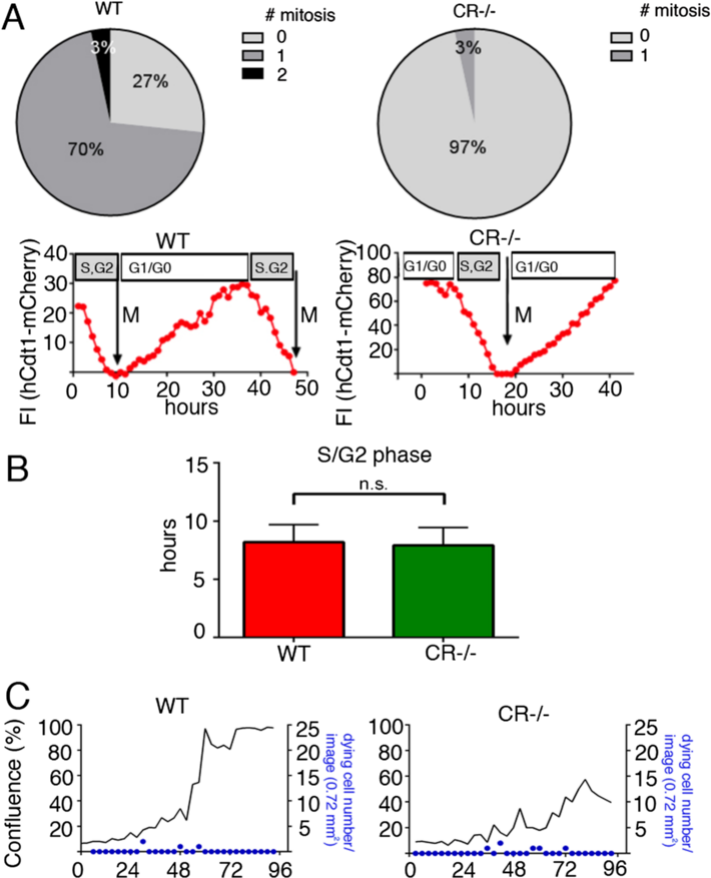
**a** During a 24 h observation period 70 % of WT prMC undergo mitosis (Mit1), 3 % of cells even 2 mitosis (Mit2) compared to CR−/− cells, where only 3 % of cells undergo mitosis (*n* = 30 cells per genotype). **b** Cell-cycle dependent changes in hCdt1-mCherry fluorescence monitored in prMC from WT and CR−/− mice. While the length of the S/G_2_/M phase is similar in both genotypes, the slower proliferation rate in CR−/− cells is the result of a prolonged G_0_/G_1_ phase. Signals are from 8 cells per genotype. Quantification of the S/G_2_/M phase in WT and CR−/− cells (bars represent means + SD; n = 10 cells per genotype). **c** Overlay of the real-time cell proliferation curves (*black lines*) and the density of dying cells (*blue*) in WT (*left*) and CR−/− (*right*) cell cultures. The blue dots display the number of newly emerging dying cells

Since closure of a scratch consists of at least 2 processes, cell mobility and cell proliferation, we next investigated cell proliferation during the wound closing time of 24 h. 73 % of WT cells underwent at least one mitosis, whereas only 3 % mitotic CR−/− cells were observed during this period (Fig. 5a). For a detailed cell cycle analyses mesothelial cells were transfected with plasmids coding for fluorescent, cell cycle-dependent marker proteins as described before [6, 18, 19]. Expression of hCdt1-mCherry is highest at the end of the G_1_ phase and decreases/disappears during the S/G_2_ phase. Thus, single mesothelial cells from WT and CR−/− mice were traced for changes in fluorescence for a period of 2 days (Fig. 5b). Since a considerable number of cells, particularly from CR−/− mice did not divide in the 2-day observation period, the precise length of the G_1_ phase could not be accurately determined. However cells entering S phase characterized by a decrease in hCdt1 fluorescence, generally entered mitosis within the observation period and thus allowed to determine the length of the S/G_2_/M phase, which was found to be approximately of 8 h, irrespective of genotype (Fig. 5b). This strongly hinted towards a prolongation of the G_0_/G_1_ phase in CR−/− cells. In order to exclude that the apparent slower proliferation results from an increase in dying cells, cell death was monitored with the Incucyte system using the CellToxGreen assay (Fig. 5c). In this assay, the cell-impermeant dye emits a strong fluorescent signal, when bound to DNA, as the result of impaired plasma membrane integrity, which occurs during necrosis and late apoptosis in vitro. After trypsinization and plating of the mesothelial cells at low density, dead (green) cells were relatively abundant in relation to the total number of cells, most probably resulting from the trypsinization. In cultures from faster growing WT cells (Fig. 5c) and in the slower growing CR−/− cells, the density of dead cells was not significantly different (Fig. 5c). Thus, the lower cell number observed in CR−/− cultures was not the result of increased cell death in the absence of CR, but most probably caused by a prolonged G_0_/G_1_ phase. In summary, several mechanisms in WT mesothelial cells contribute to a faster gap closure including higher proliferation (Figs. 2 and 5) and increased mobility (Fig. 4).

### Mesothelial cells overexpressing nuclear-targeted CR (NLS-CR) show an increased proliferation in both genotypes

**Fig. 6:**
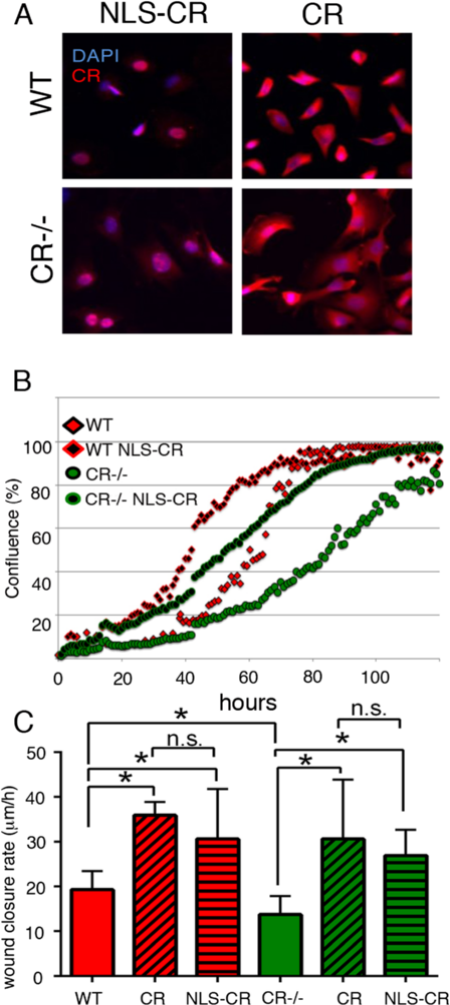
**a** IHC showing nuclear localization of NLS-CR and relatively homogenous localization of CR in WT and CR−/− prMC overexpressing either variant. **b** Expression of NLS-CR leads to an increase in proliferation as well as a decrease in the wound closure rate (**c**) in prMC from either genotype (WT and CR−/−). Effects exerted by CR and NLS-CR are similar

Although CR is considered mostly as a cytosolic protein, several reports on CR expression in cell lines in vitro and more importantly in MM samples from patients [24] have demonstrated strong nuclear CR immunolabeling. We set out to test, whether the proliferation phenotype by CR overexpression in WT and CR−/− cells could be reproduced by selectively expressing CR in the nucleus. For this we generated a plasmid coding for a protein named NLS-CR with a nuclear localization signal (NLS) sequence followed by the cDNA coding for full-length CR. Transduction by lentivirus of primary mesothelial cells from WT and CR−/− mice resulted in a strong signal in cells stained with a CR antiserum and staining was mostly confined to the nucleus (Fig. 6a). Expression of NLS-CR increased the rate of cell proliferation (left shift of the logarithmic growth phase) in both WT and CR−/− mesothelial cells (Fig. 6b). In the scratch assay NLS-CR also shortened the “wound closure time” for cells of both genotypes evidenced by the increased wound closure rate (Fig. 6c). In the same series of experiments also the wound closure rates caused by CR overexpression were measured. A significant increase in the closure rate was observed for both, CR and NLS-CR overexpressing cells in comparison to control prMC from either WT or CR-/-mice; the magnitude of the effect, i.e. the increase of the closure rate was the same for CR and NLS-CR (Fig. 6c). Thus, NLS-CR had a strong effect on proliferation and wound closure rates; this indicates that besides CR’s well-accepted function as a Ca^2+^ buffer [25] CR might have additional functions, probably also in the nucleus, possibly implicated in the regulation of transcription [26] (for more details, see Discussion).

### CR and NLS-CR affect the morphology of prMC

**Fig. 7:**
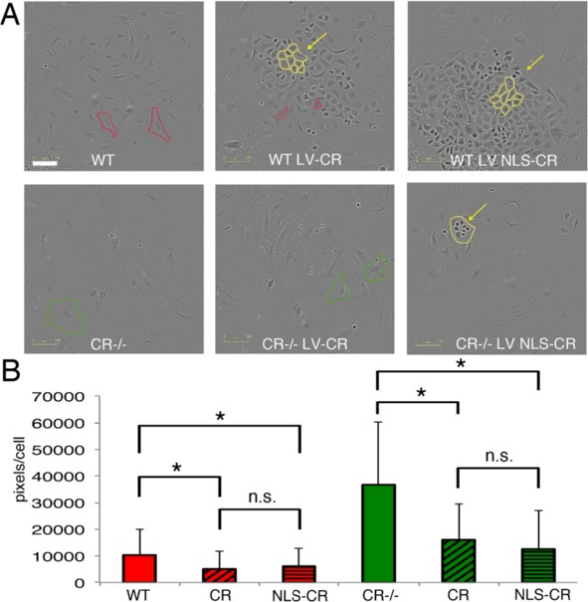
**a** Bright field images of prMC from WT and CR−/− mice taken during the rapid (logarithmic) proliferation phase. Cells are characterized by their flat morphology yielding rather low-contrast images. For better visualization, the boundaries of selected cells are marked by red (WT) or green (CR−/−) lines. The surface area of individual randomly selected cells was quantified (**b**). The surface area of CR−/− cells is clearly larger (also shown in Fig. 1j). Overexpression of CR (LV-CR) or nuclear-targeted CR (LV NLS-CR) changes the morphology to cobblestone-like cuboid cells in prMC of both genotypes (**a**). Areas containing mostly polygonal cuboid cells or rounded mitotic cells are marked by yellow lines. Surface areas covered by those cells are significantly smaller than by the parental cells (*p < 0.05). No differences exist between prMC expressing either CR variant (n.s.). Scale bar: 100 μm

Based on the differences in the surface area covered by prMC derived from WT and CR−/− mice (Fig. 1j), we tested whether overexpression of CR and NLS-CR affected the size of prMC of either genotype. WT cells were characterized by a rather flattened morphology (Fig. 7a) as also shown in Fig. 1b, c); cells from CR−/− mice covered an even larger area, also shown in Fig. 1b, c). Quantitative analyses revealed the area covered by a single CR−/− prMC to be clearly larger (Figs. 7b and 1j). Overexpression of both CR and NLS-CR was accompanied by a morphological change resulting in cells with a rather cobblestone-like morphology, typical for reactive mesothelial cells in situ, as well as for epithelioid MM cells (Fig. 7a). As a consequence, the area covered by one cell was much reduced; the size reduction was of similar magnitude for prMC overexpressing CR or NLS-CR. A size reduction was also present in CR−/− prMC overexpressing either CR variant (Fig. 7b); no statistically significant differences were evident when comparing the effects of CR and NLS-CR (Fig. 7b). Of note CR−/− prMC, even after increasing levels of CR or NLS-CR, covered a larger area than the corresponding WT prMC indicating that the overexpression of either CR variant in CR−/− prMC wasn’t sufficient to fully revert to the WT morphology phenotype.

### CR is transiently expressed during embryonic development in lung mesenchymal tissue and is correlated with the differentiation of mesothelial cells

**Fig. 8:**
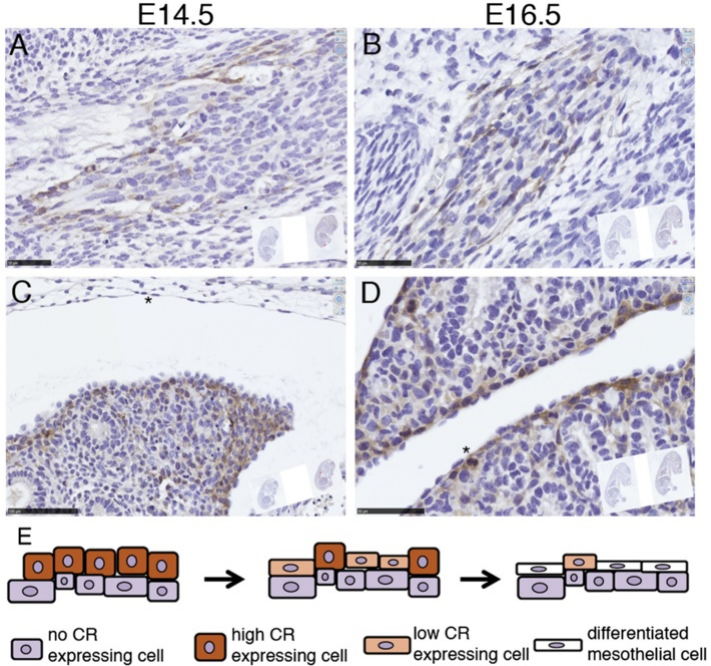
CR expression in cells of embryonic connective tissue (mesenchyme) in mouse embryos of E14.5 and E16.5 (**a**, **b**). The developing lung mesothelial precursor cells surrounding the lung (epithelial) tissue show transient expression of CR. Cuboidal proliferative cells including a mitotic cell (* in D) show high CR expression, while likely differentiated mesothelial cells (flat morphology, arrowheads in **c** & **d**) show weak to absent CR immunoreactivity (**c**, **d**). Schematic drawing of the proposed differentiation process from precursor mesenchymal cells to mesothelial cells and the corresponding transient CR expression (**e**). The proposed model is in agreement with recent findings of lineage analysis during lung mesenchyme development [43]. Scale bar 50 μm in (**a**) and (**c**) and 100 μm in (**b**) and (**d**)

Since CR protein expression levels in primary mesothelial cells of both genotypes were below the detection level of the Western blot analyses (Fig. 1g), we summoned that the observed differences between WT and CR−/− cells including decreased proliferation and wound closure time might result from differences in the development of these cells during embryogenesis. Indeed, mesothelial cells develop from mesenchyme and transient CR expression in chicken mesenchymal cells had been reported before [27, 28]. In a mouse reporter strain (Calb2^tm1(cre)Zjh^) the activity of the *Calb2* promoter was demonstrated by lacZ staining in the mesenchyme of the lung [25]. Thus, we investigated CR protein expression in the mouse lung mesenchyme and the embryonic mesothelium. Expression of CR was observed in some dispersed cells in the lung mesenchyme of E14.5 and E16.5 mouse embryos (Fig. 8a, b). In the developing lung CR-positive cells were observed in the mesenchyme surrounding the epithelial cells and the staining was particularly strong in cells forming the mesothelium or just below the layer of mesothelial precursor cells at E14.5 (Fig. 8c). At E16.5 strong staining was observed in the developing mesothelium characterized by cuboidal cells, whereas likely differentiated, i.e. mesothelial cells characterized by their flat morphology were devoid of CR expression (Fig. 8d). Proliferating, e.g. mitotic cells (asterisk in Fig. 8d) adjacent to the mesothelial cell layer also showed strong CR staining. Of note, the completely flat mesothelial cell layer of the visceral pleura indicative of a fully differentiated mesothelium already at E14.5 was CR-negative, while cuboidal cells (active, proliferating cells in development) of the parietal pleura of the lung revealed high CR expression. Thus, CR is transiently expressed in mesothelial precursor cells during mouse embryonic development and the development of the visceral pleura appears to precede the one of the parietal pleura, based on cell morphology and CR staining.

## Discussion

In this study we provide evidence that CR and moreover nuclear-targeted CR is able to increase proliferation and mobility when expressed in prMC in vitro; the presence of either variant caused a morphological change towards a more epithelioid morphology. In addition we observed that CR is expressed in mesothelial progenitor during lung development. While CR-deficient mice develop a normal mesothelium in vivo, phenotypic differences exist in CR−/− primary mesothelial cells maintained in vitro when compared to WT-derived mesothelial cells.

CR expression is considered as one of the most selective markers for mesothelioma [29]. However, CR’s function has been investigated in greater detail mostly in neurons [30], recently also describing CR’s undisputed Ca^2+^ sensor function [31]. In immortalized mesothelial cells, CR protects against asbestos-induced cytotoxicity and consequently CR up-regulation was proposed to favor mesotheliomagenesis [15]. In human mesothelioma cell lines CR is vital for cell proliferation and survival in vitro [6]. Thus, CR might emerge as a potential new target for mesothelioma therapy [6]. The precise molecular pathways, possible interaction sites and/or partners as well as exact function(s) of CR are still unknown in mesothelial cells and mesothelioma. In our study we showed the involvement of CR in the proliferation of primary mesothelial cells mimicking to some extent the reactive mesothelium, where CR expression has been observed [3].

CR protein in cultured primary mesothelial cells maintained for a short period (<8 weeks in vitro) was found to be absent or then present at extremely low levels, i.e. below the detection limit of Western blot analysis. In neurons, where CR was shown to act as a Ca^2+^ buffer [32, 33], the protein concentration is in the tens of micromolar range. The CR concentration in rat inner and outer hair cells was reported as 19 ± 2 μM and 35 ± 3 μM, respectively [34] similar as estimated in cerebellar granule cells (30 μM) [35]. In human mesothelioma cell lines, CR concentrations were found to be in the same range (1 – 100 μM) (W. Blum, unpublished data). Since in cultured primary mesothelial cells, CR levels were below the detection limit of our Western blot analyses we estimated that [CR]_i_ was below 100 nM. On the other hand, CR overexpression levels after lentivirus transduction may reach several hundreds of micromolar, as estimated by overexpression of a fusion protein consisting of the enhanced blue fluorescent protein and CR in primary mouse mesothelial cells using the identical lentiviral expression system [8]. The effects on proliferation and mobility were similar when CR overexpression was specifically nuclear. CR’s specific sensor functions in the nucleus might be of relevance, CR was previously shown to inhibit the DNA binding of E-proteins and CR overexpression inhibited activation of transcription by the E-proteins E12 and E47 [36]. For directed migration occurring during the scratch closure process, microtubular reorganization plays a pivotal role [37]. Microtubule organizing centers (MTOCs) and centrioles play an essential role in establishing the direction of cell migration [38]. In endothelial cells, the MTOC turns towards the cell-depleted space, when the cells start to migrate. Our findings may also shed light on the importance of the previously reported interaction between CR and the cytoskeletal system including the centriole in interphase cells [39, 40].

An explanation for the observed differences between WT and CR−/− primary mesothelial cells might lie in differences resulting during embryonic development. Here we showed that mesothelial precursor cells with cuboid morphology transiently expressed high levels of CR in WT mice evidenced at E14.5 and E16.5. The fact that CR is expressed transiently during lung branching morphogenesis is already exploited for conditional gene expression at this developmental period specifically in mesothelial precursors [28]. In contrast, the single cell layer of mesothelial cells characterized by their flat morphology was CR-negative. If one hypothesizes that this transient CR expression might be linked to a developmental program, its absence in CR−/− mice might result in a slightly altered/modified developmental program. This difference did not durably affect the forming of the mesothelial cell layer, since no striking differences were observed in the mesothelium of CR−/− mice in vivo. However, small long-lasting and persistent changes might be the cause for the significantly different behavior of primary mesothelial cells observed in vitro*,* where some developmental programs may be reactivated. Expression of CR and of NLS-CR (representing the situation present in human reactive mesothelium) in both genotypes resulted in increased proliferation, scratch closure rate and a more epithelioid (cuboid) morphology. Thus, also mesothelial cells of CR−/− mice that had never experienced the transient CR expression phase were receptive to increased CR levels, either when increased within the entire cell or even when selectively expressed in the nucleus.

Precursor mesothelial cells originating from CR−/− animals might have acquired compensatory/adaptive mechanism according to the concept of the Ca^2+^ homeostasome [41] to cope with the lack of CR in order to reach full differentiation towards almost “normal” mesothelial cells. This compensation might cause some irreversible changes with respect to morphology/function and those differences might be manifest only under certain experimental conditions, e.g. when comparing proliferation and mobility properties in vitro. Interestingly, this hypothesis appears to be selective for cells with a “known CR history”; fibroblasts, cells assumed to be CR-negative throughout their ontogeny are not affected, at least not with respect to parameters investigated in this study.

## Conclusions

Our results in primary mesothelial cells are in support of CR playing an important role in cell proliferation and mobility. We hypothesize that the lack of transient CR expression during embryonic development in the mesenchyme and in mesothelial precursor cells entails irreversible changes in the growth characteristics of primary mesothelial cells of CR−/− origin. The importance of CR in mesothelioma as a diagnostic marker [3, 5], as well as its essential function in mesothelioma cell lines [6] emphasize the need of understanding the function and involvement of CR in mesothelium development and mesothelium tissue repair, e.g. after exposure to asbestos. As the result of such a tissue injury, a mesothelial-mesenchyme transition might occur [42] reverting the phenotype to a CR-expressing, rapidly proliferating one. Future experiments are expected to also shed light on the question, whether CR’s absence in CR−/− mice will affect MM development in vivo.
